# PAXX/Ku interaction is rate limiting for repair of double-strand DNA breaks requiring end processing

**DOI:** 10.1016/j.jbc.2025.110481

**Published:** 2025-07-12

**Authors:** Joanna Gluza, Joanna Machnik, Malgorzata Szatkiewicz, Andrew Craxton, Steven W. Hardwick, Himani Amin, Maria M. Sąsiadek, Marion MacFarlane, Grzegorz Chodaczek, Amanda K. Chaplin, Michal Malewicz

**Affiliations:** 1 Łukasiewicz Research Network – Polish Center for Technology Development, Wrocław, Poland; 2 Wroclaw Medical University, Wrocław, Poland; 3 MRC Toxicology Unit, University of Cambridge, Cambridge, UK; 4 Department of Biochemistry, University of Cambridge, Cambridge, UK; 5 Leicester Institute for Structural and Chemical Biology, Department of Molecular and Cell Biology, University of Leicester, Leicester, UK

**Keywords:** molecular biology, DNA repair, nucleolus, DNA damage, DNA damage response, double strand break, DSB, NHEJ, non-homologous end joining, PAXX

## Abstract

In mammalian cells, DNA double strand breaks (DSBs) are primarily repaired *via* classical non-homologous end joining (c-NHEJ)—one of the most essential DNA repair pathways. As NHEJ does not utilize a template, this type of repair is the default mechanism for eliminating DSBs occurring in non-cycling cells. NHEJ is a crucial process in mammals, and defects of this repair pathway often result in immunological impairment owing to failure of somatic recombination in lymphocytes and improper neuronal biogenesis. The NHEJ machinery assembles in a stepwise process at DSBs and proceeds *via* several key repair phases including break recognition, mediated by Ku proteins (Ku70/80 heterodimer binding to DNA), DNA ends processing, and finally DNA ligation. DNA end-bound Ku recruits the large kinase protein DNA-PKcs, and downstream repair-facilitating components such as PAXX, XLF and XRCC4/Ligase IV complex that together facilitate the repair reaction. Processing of DNA breaks can require both nucleotide removal and incorporation, involving a plethora of enzymes such as nucleases and polymerases. It is currently not known which step, if any, limits the completion of the repair process. Here, we describe a single conserved amino acid substitution in PAXX protein Ku70/80 contact interface that dramatically stabilizes the repair complex. This mutation leads to co-dependent mislocalization of PAXX and Ku to the nucleoli. Surprisingly, this novel PAXX gain-of-function mutation accelerates NHEJ repair but only of DSBs that require end processing such as radiation-induced DSBs. Thus, in mammalian NHEJ, the repair complex stability is rate-limiting for the overall repair reaction of DSBs.

Double strand DNA breaks (DSBs) are considered the most toxic DNA lesions in the cell (1) threatening genome stability by physically impairing both DNA transcription and replication. In addition, with multiple DSBs, the process needs to ensure correct DNA end bridging in order to avoid chromosomal translocations. During DSB repair, the cellular machinery must protect the DNA ends from excessive processing and/or random degradation to facilitate faithful repair. Considering these threats, it is not surprising that cells have developed sophisticated DSB repair machinery. Mammalian cells utilize a number of pathways for DSBs repair that are categorized as either homologous recombination (HR) or non-homologous end joining (NHEJ), depending on whether it involves a templated (HR) or non-templated (NHEJ) repair (2). At the organismal level, the classical NHEJ is an essential process in mammals and the principal DSB repair pathway in non-cycling cells (G0/G1 phase). NHEJ does not require a DNA template as a donor of “undamaged” DNA sequence for the repair process to proceed, and can incorporate foreign DNA and/or tolerate short deletions/insertions (indels), typically up to 10 bp (3). Although DNA end processing is not necessary to join ends generated by the enzymatic activity (“clean” DSBs), pathological breaks that result from radiation exposure commonly possess blocked ends that must be rendered ligation compatible. Mechanistically, NHEJ functions by using the heterodimeric Ku70/Ku80 proteins (encoded by *XRCC6/XRCC5* genes, respectively) to enable free DNA end recognition at the break. DNA end-bound Ku adopts an altered conformation that recruits DNA-PKcs protein, which exhibits kinase activity. DNA-PKcs subsequently phosphorylates itself and helps to form a DNA end-protecting repair complex. The Ku/DNA-PKcs complex (DNA-PK) is also responsible for DNA end bridging (synapsis) by forming so-called long-range (LR) synaptic complexes. In the following steps, additional NHEJ components such as the Artemis nuclease, XLF (and XRCC4), and PAXX can be recruited to help orchestrate DNA end processing. The NHEJ holocomplex is able to adopt functionally distinct long-range synaptic structures at the break (4, 5, 6, 7, 8). Finally, once the DNA ends are compatible with ligation, the transition to a more compact short-range synaptic complex (SR) at the DSB allows Ligase IV to complete the repair process. Several recent studies have shed light on additional structural details of NHEJ complex assembly and functions (9, 10, 11). NHEJ is able to deal with a variety of DSB structures and is capable of processing individual DNA ends separately in an iterative fashion to arrive at ligation-compatible ends (12). Although it is known that the repair reaction proceeds over several consecutive stages, it is unclear whether there are any factors limiting the speed of the repair. Intuitively, one could expect that the faster the repair, the less accurate it would be. So, naturally, one could assume that a cell should favor slower repair in order to maximize the resulting accuracy. On the other hand, too slow DSB repair could increase the chance of translocations and would hamper the restart of transcription and/or replication, leading to undesired effects on cellular physiology.

PAXX is the most recently discovered component of NHEJ (6, 7, 8). Over the last decade, numerous studies highlighted important functions of PAXX in either stabilization of NHEJ at the DSBs (13), orchestration of DNA end processing (4), and synaptic complex assembly (10, 11). Notably, PAXX has been shown to be vital in the absence of XLF. However, PAXX is unable to form a stable complex with DNA alone and instead is recruited to DSBs *via* direct protein-protein interactions with the Ku70 subunit of the Ku dimer (10, 11, 14).

In this work, we have identified a novel gain-of-function PAXX K193R mutation that increases the stability of PAXX/Ku interaction and, surprisingly, shows mislocalization of PAXX protein into nucleoli. In addition, the PAXX K193R mutation dramatically accelerates the repair of radiation-induced and oxidative DSBs, which require DNA end processing. Thus, NHEJ complex stability is rate-limiting in the repair reaction *in vivo*.

## Results

### Mutation of lysine residues in PAXX

**Figure 1:**
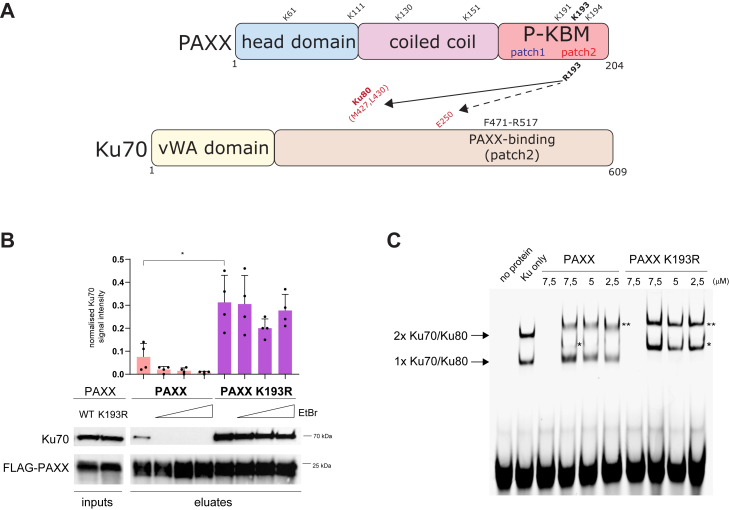
**PAXX K193R mutation leads to constitutive DNA-independent interaction with Ku70.***A*, PAXX and Ku70 proteins linear cartoons with key interacting and functional regions and all PAXX lysine (K) residues indicated. *Arrows* point to major intermolecular interactions identified in this study. *B*, immunoprecipitation of endogenous Ku70 protein with FLAG-PAXX WT/K193R variants transiently transfected into U2OS PAXX knockout cells. Indicated above the blot image, increasing amounts of ethidium bromide (EtBr). Bar graphs above the WB *panel* indicate the normalised mean signal intensity for Ku70 (n = 4; SD; ∗*p* = 0,011). Position of molecular weight marker is depicted on the right. *C*, electrophoretic mobility shift assay (EMSA) of purified Ku dimer incubated with either DNA alone, Ku only or decreasing amounts of WT/K193R recombinant PAXX protein (protein concentration is depicted above lanes in μM). *Arrows* depict DNA/Ku/PAXX complexes containing either a single Ku70/Ku80 molecule or two Ku70/Ku80 molecules bound to the probe. *Single asterisk* depicts PAXX bound to a complex with a single Ku70/Ku80 molecules. *Double asterisk* depicts PAXX bound to a complex with a two Ku70/Ku80 molecules. In lanes three and seven no Ku protein was added.

In an effort to better understand the role of PAXX, we embarked on a systematic approach to address potential lysine post-translational modifications of PAXX. We replaced all seven PAXX lysine residues with arginine to maintain their charge, herein termed PAXX 7KR (Figs. 1*A* and S1). PAXX is predominantly localized to the nucleus, with relatively little presence in the nucleoli (8). Surprisingly, PAXX 7KR mutant showed abnormal and prominent nucleolar localization (Fig. S1). This effect was specifically caused by the mutation of the K193 residue in the PAXX protein. Indeed, restoring the single lysine at position 193 within the 7KR background (7KR-R193K mutant) was sufficient to normalize the localization of PAXX (Fig. S1). Given that the K193 residue is located within the Ku-binding motif of PAXX (P-KBM; Fig. 1*A*), we assessed the interaction of the PAXX K193R mutant with endogenous Ku complexes. Interestingly, the interaction of endogenous Ku70 protein with PAXX K193R was much stronger than with PAXX WT (Fig. 1*B*) and was resistant to the DNA intercalator, ethidium bromide (EtBr), suggesting that DNA was not required to stabilize the interaction (Fig. 1*B*). Furthermore, the PAXX K193R mutant has a strong ability to supershift DNA-bound Ku (Fig. 1*C*). PAXX WT, unlike PAXX K193R, was only able to supershift DNA/Ku complex containing two molecules of Ku dimer, whereas the migration of monomeric Ku/DNA complex was relatively unaffected by the addition of PAXX WT (Fig. 1*C*), in agreement with previously published data (7).

### PAXX K193R mislocalizes Ku70/80 to nucleoli

**Figure 2:**
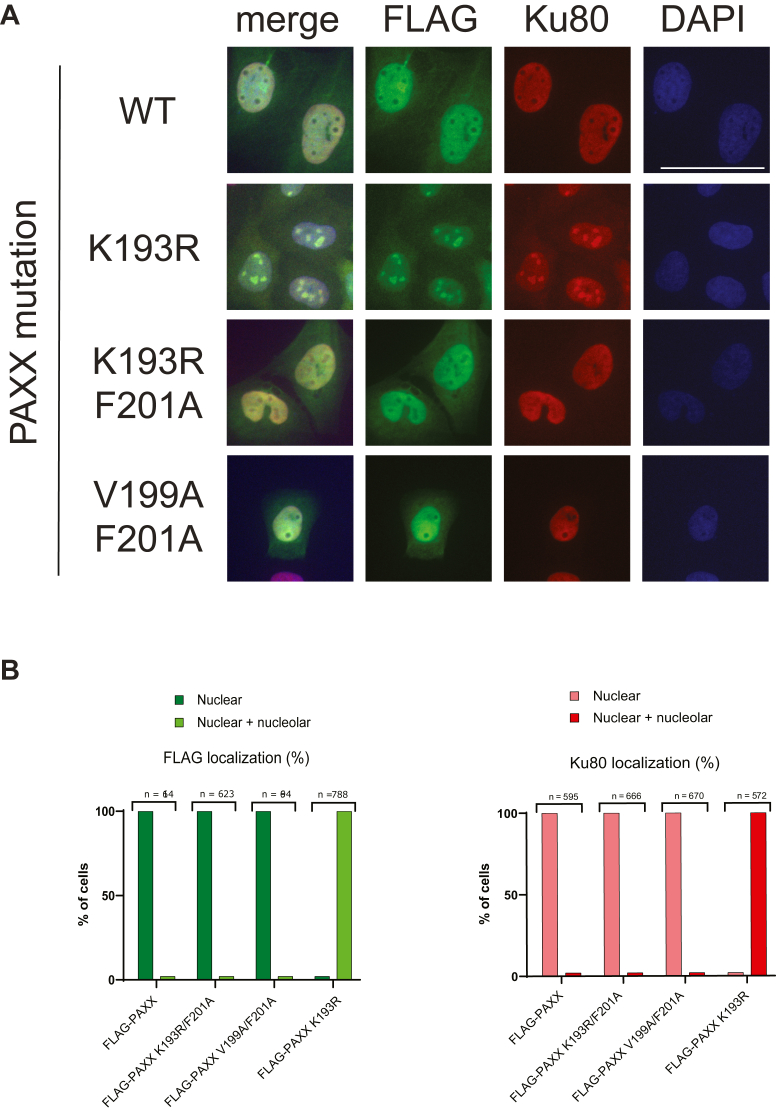
**PAXX K193R mutation results in nucleolar PAXX/Ku complex formation.***A*, immunofluorescence staining showing representative images of various PAXX mutants (FLAG-tagged; plasmid-derived) and Ku80 (endogenous) proteins in U2OS PAXX knockout background. DAPI – depicts nuclear stain. *B*, quantification of immunofluorescence experiments from *panel A*. “Nuclear” pattern is defined absence of staining in the nucleoli. “Nuclear and nucleolar” pattern is defined by the presence of staining both in the nucleus and the nucleoli. n – the number of scored nuclei; scale bar = 50 μm.

Strikingly, PAXX K193R caused Ku80 intra-nucleolar localization even in the absence of DNA damage in U2OS, HEK293, and RPE-1 cell lines (Fig. 2*A* and data not shown). Nucleolar presence of PAXX K193R was not affected by DNA damage (data not shown). To address whether the intra-nucleolar localization of the K193R mutant was a consequence of its interaction with the Ku complex, we introduced a second mutation into PAXX K193A (previously shown to disrupt PAXX/Ku interaction (7, 11)). PAXX K193R/F201A double mutant showed intra-nuclear distribution excluding nucleoli, similar to PAXX V199A/F201A double mutant used as a control (Fig. 2, *A* and *B*). Thus, the F201A PAXX mutation was dominant over the K193R substitution. Our data demonstrate that constitutive PAXX/Ku complex formation induced by the K193R mutation is responsible for the mislocalization of the complex to nucleoli.

### PAXX K193R accelerates DSB repair

**Figure 3:**
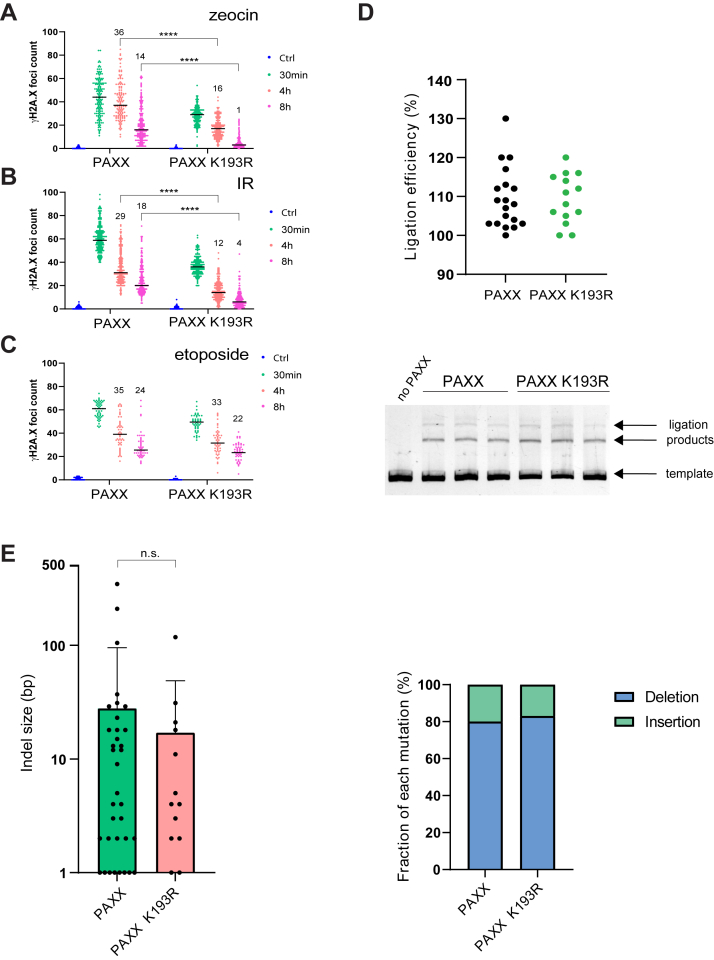
**PAXX K193R mutation accelerates repair of “blocked” DSBs.***A*–*C*, DSB repair foci count at indicated time points after exposure of U2OS PAXX KO cells expressing either PAXX WT or PAXX K193R protein to zeocin, X-rays (IR) or etoposide. (Ctrl) denotes the number of observed DSB foci prior to exposure to DNA-damaging agent (for *A* and *B* n = 3, median, ∗∗∗∗*p* < 0,001; for *C* n = 2, median). Numbers above 4 h and 8 h data points indicate median number of unrepaired foci. *D*, *in vitro* DNA ligation assays. The graph shows quantification of ligation reactions from three independent experiments (n = 3), where 100% was the level of ligation observed in the absence of PAXX. The gel picture in the *lower panel* shows a representative migration of the ligation products and the non-ligated template DNA. *E*, *leftpanel* shows the quantification of mean indel size (n = 3, SD; n.s. denotes statistically not significant) whereas *right panel* depicts proportion of deletions *versus* insertions observed after repair of CRISPR-induced chromosomal DSBs (n = 3).

Next, we assessed the impact of the K193R mutation on the double strand DNA break repair process. We induced DSBs *via* oxidative DNA damage (Figs. 3*A* and S2*A*) or a low dosage of gamma irradiation (Figs. 3*B* and S2*B*) and counted γH2A.X positive foci (DSB markers) in cell nuclei. Surprisingly, while cells expressing PAXX WT protein still showed substantial amounts of unrepaired DSBs at 8 h time point post treatment, the PAXX K193R-expressing cells had almost completely repaired the damage. Strikingly, already at 30 min post-irradiation there were substantially fewer DSBs visible in the K193R PAXX mutant, which likely reflects the accelerated repair occurring prior to visible DSB foci development (typically requiring 15–20 min post induction). Thus, K193R mutation in PAXX dramatically accelerated DSB repair process *in vivo*. Zeocin and IR-induced DNA damage is known to result in so-called “dirty” DNA ends that require removal of damaged bases and nucleotides (processing) before ligation to complete the repair. Thus, we utilized etoposide, a topoisomerase II (TopoII) poison, that traps TopoII-DNA complex and results in blunt (“clean”) ends, which can be directly repaired without prior processing (apart from the need of removal of covalent TopoII protein-DNA crosslink by specialized enzyme TDP2 (15)). Strikingly, PAXX WT and K193R showed a similar overall repair efficiency of etoposide-induced breaks (Fig. 3*C*) suggesting that the impact of K193R mutation is restricted to DSBs requiring DNA end processing. In agreement with this hypothesis, both the indel structure of DSBs repaired after Cas9 nuclease cutting (Fig. 3*E*) and *in vitro* ligation reactions with purified NHEJ proteins on blunt-ended substrates (Fig. 3*D*) did not reveal any differences between the PAXX WT and PAXX K193R proteins.

### Structural characterization of PAXX K193R mutation

**Figure 4:**
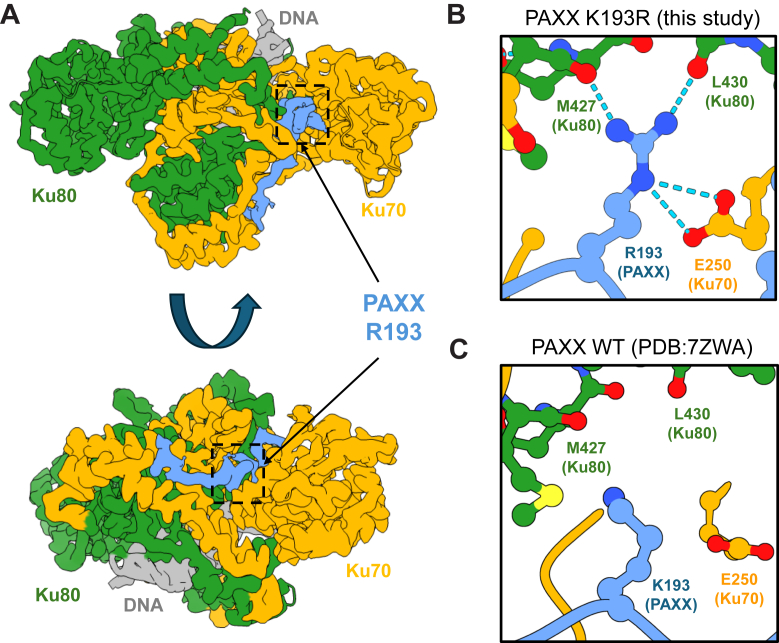
**Cryo-EM structure of Ku70/80 with the PAXX peptide mutant K193R.***A*, cryo-EM map of Ku70/80 with the PAXX peptide mutant K193R to 2.8 Å resolution. Ku70 is in *yellow*, Ku80 in *green*, DNA in *gray*, and the PAXX peptide in *blue* with an *arrow* indicating the location of the mutated residue. *B*, enlarged inset of the R193 residue shown in *blue*, interacting with the backbone oxygens of Ku80 shown in *green*, and E250 of Ku70 *C*) Enlarged inset of the PAXX WT, K193 residue shown in *blue* with surrounding Ku80 residues labeled and shown in *green*.

In an effort to provide the molecular basis for the observed phenotypes, we collected cryo-EM data of the Ku70/80 heterodimer in complex with a peptide corresponding to the PAXX KBM (P-KBM) domain containing the K193R mutation. The resulting structure could then be compared to the equivalent structure of Ku70/80 in complex with wild-type P-KBM peptide (PDB:7ZWA) (11). A map of Ku70/80 in complex with P-KBM K193R was produced at 2.8 Å global resolution (Fig. 4*A*) (Figs. S3 and S4; Table S1). The structure demonstrates an increased hydrogen bonding network between the arginine side chain and Ku70/80 when compared to the wild-type (WT) structure. Specifically, the guanidinium group of R193 in P-KBM K193R forms hydrogen bonds with carbonyl oxygens of Ku80 residues: M427 and L430. Additionally, E250 of Ku70 is within bonding distance of the R193 side chain of P-KBM K193R (Fig. 4, *B* and *C*). We exploited the finding that PAXX specifically interacts with the Ku70 protein (14). Co-immunoprecipitation experiments showed that PAXX makes electrostatic interactions with E250 side chain (depicted with arrows on Fig. 1*A*) (Fig. S5), as revealed by a E250A severe reduction in interaction with both WT and PAXX K193R. Considering that arginine side chain electrostatic interactions with either glutamic or aspartic acid residues are at least twice as strong energetically as corresponding lysine interactions (16), it likely contributes to explaining why substitution of lysine with arginine at position 193 in PAXX affects the Ku70 interaction. Strikingly, R193 is able to reach Ku80 by forming carbonyl-H bonds, to stabilize the overall PAXX K193R/Ku dimer interaction.

In summary, we described the first ever gain-of-function mutation in DNA repair that accelerates DSB repair, revealing the presence of rate-limiting steps in the overall repair process.

## Discussion

It had been observed that the NHEJ machinery is excluded from the nucleoli (8). The reason and the mechanism are unknown; however, the rationale might relate to the fact that nucleoli possess multiple repetitive clusters of rDNA. Repair of DSB damage in such repetitive elements *via* NHEJ might lead to genome scrambling thus NHEJ activity in nucleoli could be undesired. In fact, it had been suggested that rDNA DSB repair mobilizes HR even in the G1 phase of the cell cycle (17). We would like to propose that PAXX (and other NHEJ factors) are actively exported from the nucleoli. Given that PAXX K193R strongly interacts with Ku complex even in the absence of DNA (Fig. 1*B*), it appears that the pre-formation of NHEJ-like repair complex in the nucleolus is the reason for the retention of these factors in that compartment. The co-dependence of PAXX/Ku interaction on the nucleolar retention of PAXX K193R mutant protein might suggest that larger assemblies fail to efficiently exit nucleolus. Clearly, both the reason and the mechanism of NHEJ nucleolar exclusion deserve further attention.

In recent years, relatively little attention has been devoted toward the understanding of possible bottlenecks of the DSB repair reaction, *i.e,* the existence of possible rate-limiting steps. The NHEJ repair complex needs to stably associate with DSBs within the chromatin complex, fending antagonistic activities from other DSB sensors such as the MRN complex or PARPs (18). At the same time, the flexibility of the NHEJ repair reaction demands structural rearrangements of the assembled complex to reflect the ongoing end processing. It is possible that NHEJ machinery might fall off the chromatin during the repair, which will require *de novo* assembly at a given DSB. Thus, the machinery needs to balance the flexibility (loose fit) with the demand of maintaining end association (rigid fit). Clearly, the control points for this regulation are the interaction surfaces between the NHEJ components. Recently, there has been a lot of interest in so-called KBMs (Ku-binding domains; (19)). KBMs are short conserved amino acid stretches found in DNA repair factors that utilize Ku as their main recruitment modality. Here, we demonstrate that stabilizing one of the PAXX KBMs (P-KBM) has a dramatic effect on the repair process. The repair of so-called “blocked” DSBs is special in that it requires end processing to create ligatable DNA end configurations. We notice a very good fit of the R193 PAXX mutant residue into a groove formed between Ku70 and Ku80. Does that suggest a regulatory modification occurring naturally at the K193 PAXX residue? Indeed, we are currently investigating this hypothesis. Why is the stabilization of Ku70/PAXX interaction leading to accelerated repair of such DSBs? We hypothesize that either Ku might have a tendency to fall off the chromatin during the end processing phase, or artificially stablised Ku70/PAXX complex prevents utilization of alternative repair pathways, leading to a more linear and potentially faster repair mechanism. Accurate measurements of Ku70 DSB off-rates could help explain the effect of the K193R mutation. Studying the mechanism of repair elicited by the K193R mutation might shed light on the likelihood of the second explanation. The existence of a pre-assembled NHEJ complex might also be possible.

The propensity of R193 PAXX K193R residue to reach out to Ku80 (Ku70 is the natural PAXX interactor) raises evolutionary implications, as to whether the complex was evolving towards a weaker (more flexible and preferential from the regulatory perspective) structure.

Another possible effect of the K193R mutation is on the synapses of DNA ends (DNA end bridging). As revealed by our EMSA experiments, PAXX K193R is able to supershift monomeric Ku70 bound to DNA (Fig. 1*C*). Extrapolating this observation to the repair *in vivo* suggests that PAXX K193R could stretch across the break much more efficiently than PAXX WT. It would be interesting to assess the effect of the mutation on translocation frequencies. We also expect PAXX K193R mutant to show some trade-offs, for example, faster repair might mean shorter or longer (or more frequent) indels, depending on what occurs first on the route to ligatable ends. It is currently technically unfeasible to establish the sequence of indels arising from the repair of oxidative DSBs, as they occur randomly in any exposed cell.

Our data open a way for more systematic DNA repair pathway engineering, as already well underway elsewhere, revealed by recent reports on the evolution of HR and NHEJ efficiency (20, 21). Faster NHEJ could benefit both biotechnology (mutant generation *via* CRISPR) as well as medicine (possible radioprotection). For now, we conclude that the PAXX K193R mutation is not ideally suited for such purposes, as it renders PAXX/Ku interaction constitutive due to the fact that the DNA-dependent aspect of the regulation is lost. In addition, the PAXX K193R-associated mislocalization of NHEJ components to nucleoli is also undesirable. Further mutagenesis of the now well-defined PAXX/Ku interface might produce more suitable “fast” NHEJ variants.

Our original rationale to replace the PAXX lysine residues with arginines was to prevent lysine-dependent post-translational modifications (such as ubiquitination, for example), maintaining the positive charge in that specific position. Clearly, alterations of K193 to A193 or D/E193 would also be interesting avenues for future investigation, although loss of positive charge in the PAXX 193 position is likely to weaken interaction with Ku70, potentially producing a partial loss-of-function effect. PAXX K193R mutation shows that even very minor chemical alterations could in fact, have a major functional impact.

## Experimental procedures

### Cell culture and transfection

HEK293H (Invitrogen) and U2OS (ATCC) cells were cultured in DMEM medium containing 4.5 g/L D-glucose, GlutaMAX, and either 1 mM sodium pyruvate (HEK293H) or no pyruvate (U2OS), supplemented with 10% (v/v) FBS (Merck). RPE-1 cells were grown in DMEM/F-12 (1:1) medium containing L-glutamine and 15 mM HEPES supplemented with 10% FBS. Suspension-adapted HEK293F (Invitrogen) cells were cultured in FreeStyle 293 Expression Medium (Invitrogen). All cell cultures were grown at 37 °C with 5% CO_2_ (adherent cells) or 8% CO_2_ saturation and 125 rpm shaking (suspension-adapted cells).

Transfections were performed according to the manufacturer’s instructions. U2OS cells were transfected with Lipofectamine 2000 (ThermoFisher Scientific), while Lipofectamine LTX and FreeStyle MAX reagent (ThermoFisher Scientific) were used for HEK293H and HEK293F cell transfection, respectively.

### Generation of FLAG-PAXX and FLAG-PAXX K193R U2OS cell lines

U2OS cell lines stably expressing FLAG-PAXX WT or FLAG-PAXX K193R were generated by co-transfection of U2OS PAXX knockout cells (4) with pCMX-encoded N-terminal FLAG-tagged proteins and pTKHyg plasmids at a 6:1 ratio. Upon hygromycin B selection (200 μg/ml, VWR), individual antibiotic-resistant clones were isolated and screened for the presence of both the FLAG tag and PAXX by immunoblotting.

### Immunofluorescence staining

3·10^4^ cells per well were seeded on Nunc Lab-Tek II CC2 (ThermoFisher Scientific) 8-well chamber slides and incubated overnight at 37 °C to allow cell adhesion. Upon fixation in 4% (v/v) paraformaldehyde for 10 min at room temperature, the cells were permeabilized and blocked with blocking buffer (0.3% (v/v) Triton X-100, 5% (v/v) goat serum in cold PBS) for 30 min at 4 °C. Next, the cells were incubated overnight at 4 °C with the appropriate primary antibody: FLAG (1:250; Cell Signaling Technology), Ku80 (1:50; Invitrogen, clone 111, cat. MA5-12933), γH2A.X (1:1000; Abcam), followed by 1 h incubation at room temperature with secondary antibodies and 1 μg/ml DAPI (VWR) in blocking buffer. Cells were visualized using Zeiss Cell Observer SD confocal microscope (Carl Zeiss) and analyzed with ImageJ software. Representative images shown; at least three biological repeats were acquired.

For analysis of γH2A.X foci following seeding, the cells were incubated for 1 h either with 50 μM etoposide (Sigma-Aldrich) or 50 μg/ml zeocin (InvivoGen) or alternatively exposed to 2 Gy gamma irradiation (XStrahl CIX3 cabinet) and allowed to recover for 30 min, 4 h and 8 h. Median number of unrepaired foci (shown on Fig. 3, *A*–*C*) was calculated by subtracting median number of basal foci observed prior of DNA damage induction form the median number of foci counted at 4 h and 8 h post DNA damage induction. In the initial experiments, nuclei were also costained with anti-53BP1 antibodies (Sigma-Aldrich; MAB3804) to assure that foci counted represented genuine DSBs.

## Data availability

All structural data presented are publicly available. Cryo-EM structure and map are deposited at the PDB and EMDB with accession codes as follows: PDB: 9GYF, EMDB: EMD-51697. If all data are contained within the manuscript. Please direct all request for additional data sharing to A.K.C. (regarding Cryo-EM analysis) or M.M.

## Supporting information

This article contains supporting information (4, 22).

## Conflict of interest

The authors declare that they have no conflicts of interest with the contents of this article.
