# Supplementary material for: PAXX/Ku interaction is rate limiting for repair of double-strand DNA breaks requiring end processing
Source: J Biol Chem. 2025 Jul 12;301(8):110481. doi: 10.1016/j.jbc.2025.110481 (PMC12357295; doi:10.1016/j.jbc.2025.110481)
Supplement: Supporting Figures and Tables [file mmc1.pdf]

Supporting Information

PAXX/Ku interaction is rate-limiting for repair of double-strand DNA breaks requiring end processing.

Joanna Gluza, Joanna Machnik, Malgorzata Szatkiewicz, Andrew Craxton, Steven W. Hardwick, Himani Amin, Maria M. Sąsiadek, Marion MacFarlane, Grzegorz Chodaczek, Amanda K. Chaplin and Michal Malewicz

List of Contents

DETAILED METHODS

Figure S1. PAXX K193 residue as the main nucleoli localization determinant.

Figure S2. Representative immunofluorescence images of γ-H2A.X foci used for the quantifications shown in Figure 3A and 3B.

Table S1. Cryo-EM data collection parameters and statistics.

Figure S3. Single-particle cryo-EM image processing workflow for Ku70/80-DNA with PAXX peptide K193R mutant.

Figure S4. Cryo-EM data of Ku70/80-DNA with PAXX mutant peptide K1943R.

Figure S5. Analysis of PAXX/Ku70 interaction by co-immunoprecipitation experiments with HA-PAXX WT/K193R and FLAG-Ku70 WT/E250.

DETAILED METHODS

Purification of FLAG-tagged NHEJ proteins

Following a brief wash in cold PBS, HEK293F cells were lysed for 15 min on ice in a hypotonic buffer (10 mM HEPES pH 7.9, 10 mM KCl, 0.1 mM EDTA, 0.1 mM EGTA supplemented with complete Mini Protease Inhibitor Cocktail (Roche), 1 mM DTT, 10 µM MG132 (MedChemExpress), 0.1 µM Na_3VO_4 (POL-AURA), 0.4 µM PMSF (Sigma Aldrich)). Upon addition of 0.5% (v/v) NP-40 (Sigma-Aldrich) and short vortexing, the lysate was centrifuged at 4°C, 4000 x g for 5 min and the washing step was repeated as above. Released nuclei were subsequently resuspended in a high salt buffer (20 mM HEPES pH 7.9, 420 mM NaCl, 1.5 mM $MgCl_2$, 20% (v/v) glycerol, supplemented with above-mentioned inhibitors) and incubated at 4°C for 15 min with rotation. Following centrifugation (4°C, 15000 x g, 30 min), protein extracts were incubated with ANTI-FLAG M2 Affinity Gel (Merck) beads at 4°C for 2 h with rotation. Unbound proteins were subsequently removed by centrifugation (4°C, 1000 x g, 3 min) followed by five washing steps in 50 mM Tris-HCl pH 8, 1 M NaCl, 0.2% (v/v) NP-40, 1 mM DTT. Agarose-bound proteins were released with elution buffer (50 mM Tris-HCl pH 8, 150 mM NaCl, 0.2% (v/v) NP-40, 5% (v/v) glycerol, 1 mM DTT and 150 µg/mL 3xFLAG-peptide (Merck)) and were subsequently concentrated on Amicon Ultra Centrifugal Filter (Merck).

Construct design and purification of GST-tagged NHEJ proteins

Generation of pGEX-6P-1-PAXX construct was already described elsewhere (4). pGEX-6P-1-PAXX-K193R construct was created by *BamHI*/*XhoI*-mediated excision of

PAXX-K193R insert from pCMX-FLAG-PAXX-K193R and subsequent subcloning into pGEX-6P-1 vector pre-digested with the same restriction enzymes.

Expression and purification of GST-tagged PAXX proteins in *E. coli* was performed according to the protocol described earlier in (4).

PAXX-Ku binding assay

Approximately $5 \cdot 10^6$ U2OS cells were plated on Ø10 cm dishes and either transfected the next day with FLAG-PAXX WT/K193R plasmids (transient transfection of U2OS PAXX knockout cells) or harvested by trypsinization (U2OS PAXX knockout cell line stably expressing FLAG-PAXX WT/K193R). Cells were lysed using RIPA buffer (10 mM Tris-HCl pH 8, 1 mM EDTA, 140 mM NaCl, 2.5 mM sodium deoxycholate, 1% (v/v) Triton X-100, 0.1% (v/v) SDS) supplemented with 1 mM DTT, 1x PIC (Thermo Fisher Scientific), 100 nM MG132, 1 mM Na_3VO_4, 100 nM PMSF, followed by 15 minutes incubation on ice and subsequent sonication. M2 FLAG or EZview Red Anti-HA beads (Sigma Aldrich) were equilibrated according to manufacturer's instructions, washed with glycine-HCl pH 2 buffer and blocked in 3% BSA (Sigma Aldrich) just prior application of cell lysates (if perchance with addition of ethidium bromide (Sigma Aldrich) at a final concentration of 0, 50, 100, 200 µg/mL). After 1h incubation at 4°C, bound proteins were eluted at 4°C for 1h (FLAG-beads) or 30 min (HA-beads) with 150 mM FLAG or HA-peptide (Sigma Aldrich) which were diluted in elution buffer (50 mM Tris-HCl pH 8, 150 mM NaCl, 0.2% (v/v) NP40/IGEPAL, 5% (v/v) glycerol, 1 mM DTT). Samples were analyzed using SDS-PAGE and Western Blotting with 10% of inputs and 30% of eluates loaded on a gel. Representative gel images shown, at least three biological repeats were acquired. Quantification of Ku70 and FLAG (PAXX) bands on the immunoprecipitated membrane was performed using ImageLab (BioRad). Each

lane was limited, bands were individually marked and their profile was adjusted to minimize blurring. From the report generated by the program, Adjust Total Band Volume was selected as the parameter compared – the mean of three biological replicates, Ku70:FLAG(PAXX) ratio, percentage value in reference to FLAG-PAXX-WT and standard deviations were calculated by formulas in Excel (Microsoft).

Electrophoretic mobility shift assay (EMSA)

ATTO700-labelled 50 bp dsDNA probe was prepared by hybridizing two complementary DNA fragments (Table S2). The assay was performed in a reaction buffer containing 20 mM Tris-HCl pH 7.5, 50 mM KCl, 5% (v/v) glycerol, 0,1 mM DTT, 10 µg/mL BSA with 50 nM probe and varying protein concentrations. Samples were incubated at 37°C for 30 min and were subsequently resolved on a 5% poliacrylamide gel in 0.5 x TBE buffer at 4°C. Protein concentrations used in the assay: Ku70/80 0,05 µM; PAXX 2,5-7,5 µM. Gel imaging was performed using ODYSSEY CLx Infrared Imaging System (LI-COR). Representative gel image shown, at least three biological repeats were acquired.

DNA *in vitro* ligation assay

SmaI-linearized (New England BioLabs) pUC19 plasmid was purified using QIAquick PCR Purification Kit (QIAGEN). Ligation mixtures were prepared by combining 50 ng of the digested plasmid with the indicated quantities of proteins (all reactions contained Ku70/Ku80 at 0,05µM and LigIV/XRCC4 complex at 2,5 µM; PAXX was added at 1-4 µM but we observed a saturation of PAXX addition at 1 µM) in 20 µL of reaction buffer (25 mM Tris-HCl pH 7.5, 150 mM KCl, 1 mM $MgCl_2$, 1 mM DTT, 10 µM ATP, 10% (w/v)PEG 8.000, 10 µg/mL BSA). Following 5 min pre-incubation at 37°C, XRCC4/LIG4

complexes were added and the mixtures were incubated for another 30 min. Subsequently, ligation was terminated for 30 min at 50°C with 2 µL of a reaction-stop solution (100 mM EDTA, 0.1% (w/v) SDS) and 0.2 µL of 20 mg/mL proteinase K (ThermoFisher Scientific). Samples were then loaded onto 0.8% (w/v) agarose gel in TAE buffer, pre-stained with GelRed Nucleic Acid Stain (Merck Millipore) and visualized using ChemiDoc Imaging System (Bio-Rad). Signal intensity was quantified using ImageJ software.

Analysis of DNA repair accuracy

$2 \cdot 10^6$ cells were plated on Ø6 cm dishes and grown overnight at 37°C. The next day cells were transfected with 10 nM tracrRNA (Horizon), 10 nM LYAR crRNA (Horizon) and 2 µg Cas9 to introduce indels. Following puromycin selection (5 µg/mL, InvivoGen), cellular pellets were collected to extract genomic DNA. Subsequently, isolated DNA served as template to amplify approx. 250 bp fragment of LYAR using gene-specific primers. The obtained PCR product was then cloned into pJET1.2 vector (ThermoFisher Scientific) according to manufacturer's instructions and transformed into competent *Top10* cells. The presence of indels introduced by Cas9 was verified via Sanger sequencing of plasmid DNA derived from several individual colonies. For PAXX WT 62 clones and for PAXX K193R 70 clones were sequenced (3 biological repeats, 20-25 per repeat).

Cloning and site-directed mutagenesis of PAXX and Ku70

All constructs containing either mutated PAXX or Ku70 were generated via site-directed mutagenesis (QuikChange, Agilent Technologies). pCMX-PAXX-K193R/F201A, pCMX-HA-PAXX-K193R, pCMX-FLAG-Ku70-E250A, constructs were

obtained by either Q5- (New England Biolabs) or Pfu Plus-mediated (Eurex) amplification with primers bearing point mutations (Table S2) and pCMX-FLAG-PAXX-K193R, pCMX-HA-PAXX and pCMX-FLAG-Ku70 plasmids used as templates respectively. Followed by *DpnI* digestion (New England Biolabs), nicked PCR products were transformed into competent *DH5α* cells. Constructs integrity as well as the presence of desired mutations were confirmed by Sanger sequencing.

Statistical analysis

Statistical analysis was conducted with the aid of GraphPad Prism software. Statistical significance was assessed by two-tailed t-test; “n” value reported in figure legends denotes the number of independent biological repeats for each condition.

Cryo-EM sample preparation of Ku70/80:PAXX K193R

Proteins were concentrated using a Centricon (Amicon) with a 30 kDa cut-off and buffer exchanged into 20 mM HEPES, pH 7.6, 200 mM NaCl, 0.5 mM EDTA, 2 mM MgCl2, 5 mM DTT. Purified full-length Ku70/80 was then first mixed with 15 bp 5’ overhang DNA (Table S2) then the PAXX K193R mutant peptide at a ratio 1:1.2:2, respectively.

Cryo-EM grid preparation

Aliquots of 3 µl of ~2.5 mg/ml of Ku70/80, DNA and the peptide were mixed with 8 mM CHAPSO to eliminate particle orientation bias (final concentration, Sigma) before being applied to Holey Carbon grids (Quantifoil Cu R1.2/1.3, 300 mesh), glow discharged for 60 sec at current of 25 mA in PELCO Easiglow (Ted Pella, Inc). The grids were then blotted with filter paper once to remove any excess sample, and plunge-frozen in liquid

ethane using a FEI Vitrobot Mark IV (Thermo Fisher Scientific) at 4 ^{o}C and 95 % humidity.

Cryo-EM data acquisition

The data was collected on a Titan Krios equipped with a Gatan K3 direct electron counting detector at the University of Leicester. All data collection parameters are given in Table S1.

Cryo-EM Image processing

The classification process for the cryo-EM map is summarized schematically in Figure S2. The final reconstructions obtained had overall resolutions (Table S1), which were calculated by Fourier shell correlation at 0.143 cut-off.

Cryo-EM structure refinement and model building

The model of Ku70/80 + PAXX WT (PDB:7ZWA) was used as an initial template and rigid-body fitted into the cryo-EM density. The PAXX peptide was then mutated at position 193 from a Lys residue to Arg and the model manually fitted and refined in Coot; Finally, the model was refined using Phenix real-space refinement (22).

Table S2. Oligonucleotide/peptide sequences

Name	Sequence (5'-3')	Function
**DNA oligo 50-bp labelled**	ATTO700-TAAATGCCAATGCTGCTGATACGTACTCGGACTGATTCGGAACTGTAACG	**EMSA probe (7)**
**DNA oligo 50-bp**	CGTTACAGTTCCGAATCAGTCCGAGTACGTATCAGCAGCATTGGCATTTA	
**PAXX-F201A_F**	GCTGGTGGCGTGGACGCCGATGAGACCTGACT	**Site-directed mutagenesis (this study)**
**PAXX-F201A_R**	AGTCAGGTCTCATCGGCGTCCACGCCACCAGC	
**PAXX-K193R_F**	CCGGGTTCAAGAGTAGGAAACCAGCTGGTGG	
**PAXX-K193R_R**	CCACCAGCTGGTTTCCTACTCTTGAACCCGG	
**Ku70-E250A_F**	GTTCGCGCCAAGGCGACCAGGAAGCGA	
**Ku70-E250A_R**	TCGCTTCCTGGTCGCCTTGGCGCGAAC	
**5'overhang DNA Forward**	GATCCCTCTAGATAT	**Cryo-EM 5' 15 bp DNA sequences**
**5'overhang DNA Reverse**	CGGATCGAGGGCCCGATATCTAGAGGGATC	
**PAXX R193 Mutant peptide sequence**	Biotin-RRRCPGESLINPGFKSRKPAGGVDFDET	

Figure S1. PAXX K193 residue as the main nucleoli localization determinant. Representative immunofluorescence images of various PAXX protein variants transiently expressed in PAXX knockout U2OS cells. Control – represents untransfected cells. 7KR – PAXX mutant in which all lysine residues were replaced with arginines. 7KR-R193K – PAXX 7KR mutant in which R193 residue was "switched back" to a lysine.

Figure S2. Representative immunofluorescence images of γ-H2A.X foci used for the quantifications shown in Figure 3A and 3B.

Approximate location of nuclei is depicted with dashed white line (please note that anti-γ-H2A.X antibodies used show a variable cytoplasmic background, which is not affected by DNA damage induction and does not interfere with foci quantification).

Table S1. Cryo-EM data collection parameters and statistics.

Figure S3. Single-particle cryo-EM image processing workflow for Ku70/80-DNA with PAXX peptide K193R mutant. Schematic showing particle picking using and processing including 2D classification and *ab initio* reconstruction using CryoSPARC. The main class generated with the corresponding number of particles is shown and the final map following non-uniform refinement with resolutions for an FSC of 0.143 is given.

Figure S4. Cryo-EM data of Ku70/80-DNA with PAXX mutant peptide K1943R. A) Example cryo-EM micrograph. B) Examples of 2D classes. C) Angular distribution calculated in cryoSPARC for particle projections shown as a heat map. D) FSC resolution curves and viewing distribution plot. E) Local resolution map of the Ku-DNA with PAXX K193R mutant peptide cryo-EM map. The colors corresponding to each resolution are displayed on the specific key chart below the maps.

Figure S5**.** Analysis of PAXX/Ku70 interaction by co-immunoprecipitation experiments with HA-PAXX WT/K193R and FLAG-Ku70 WT/E250.

Western blot images showing the levels of FLAG-Ku70 and HA-PAXX in extracts from transiently transfected U2OS PAXX KO cells. Position of molecular weight marker (in kDa) is depicted on the the left.

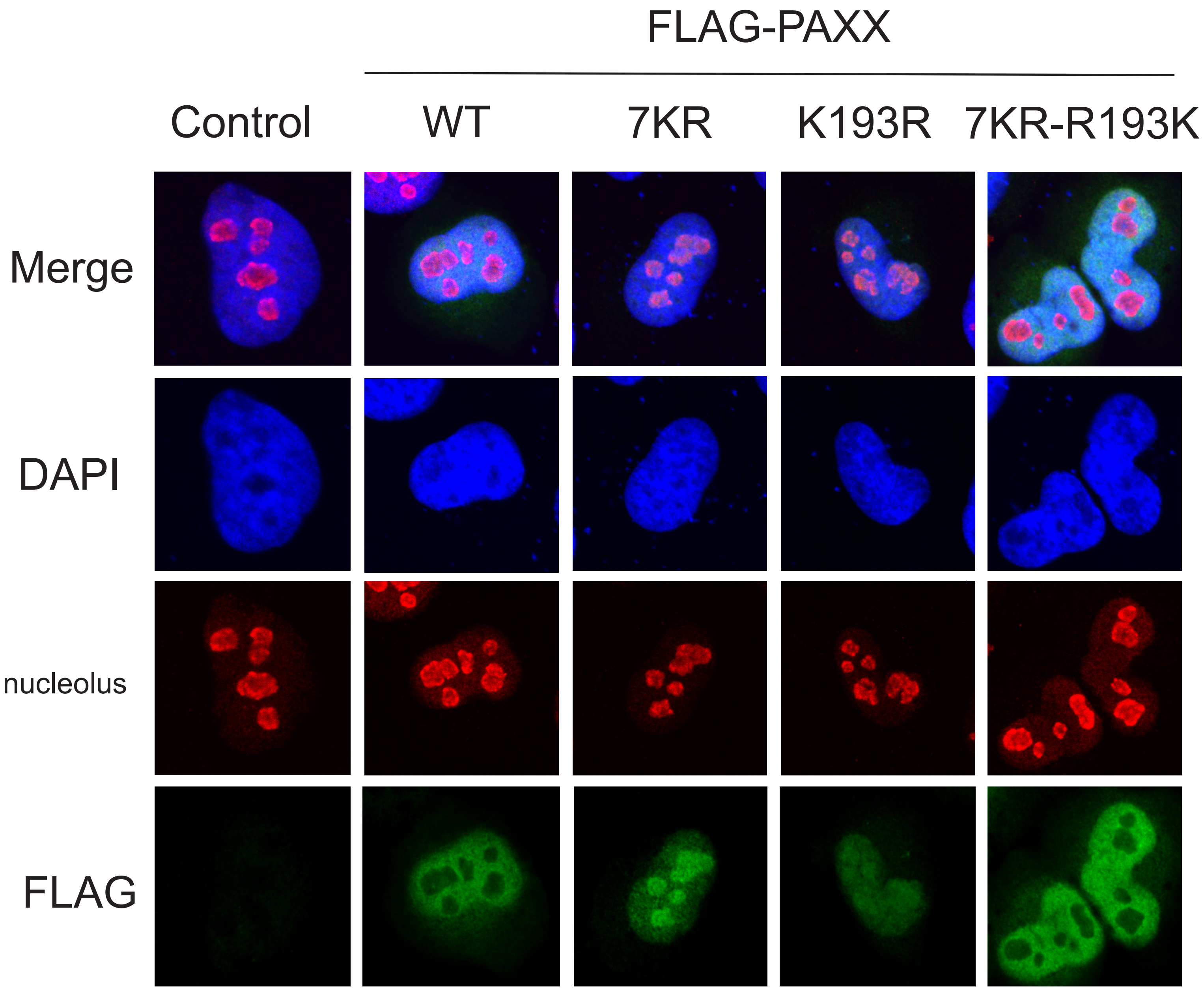
FLAG-PAXX
Control
WT
7KR
K193R
7KR-R193K
Merge
DAPI
nucleolus
FLAG

Figure S1.

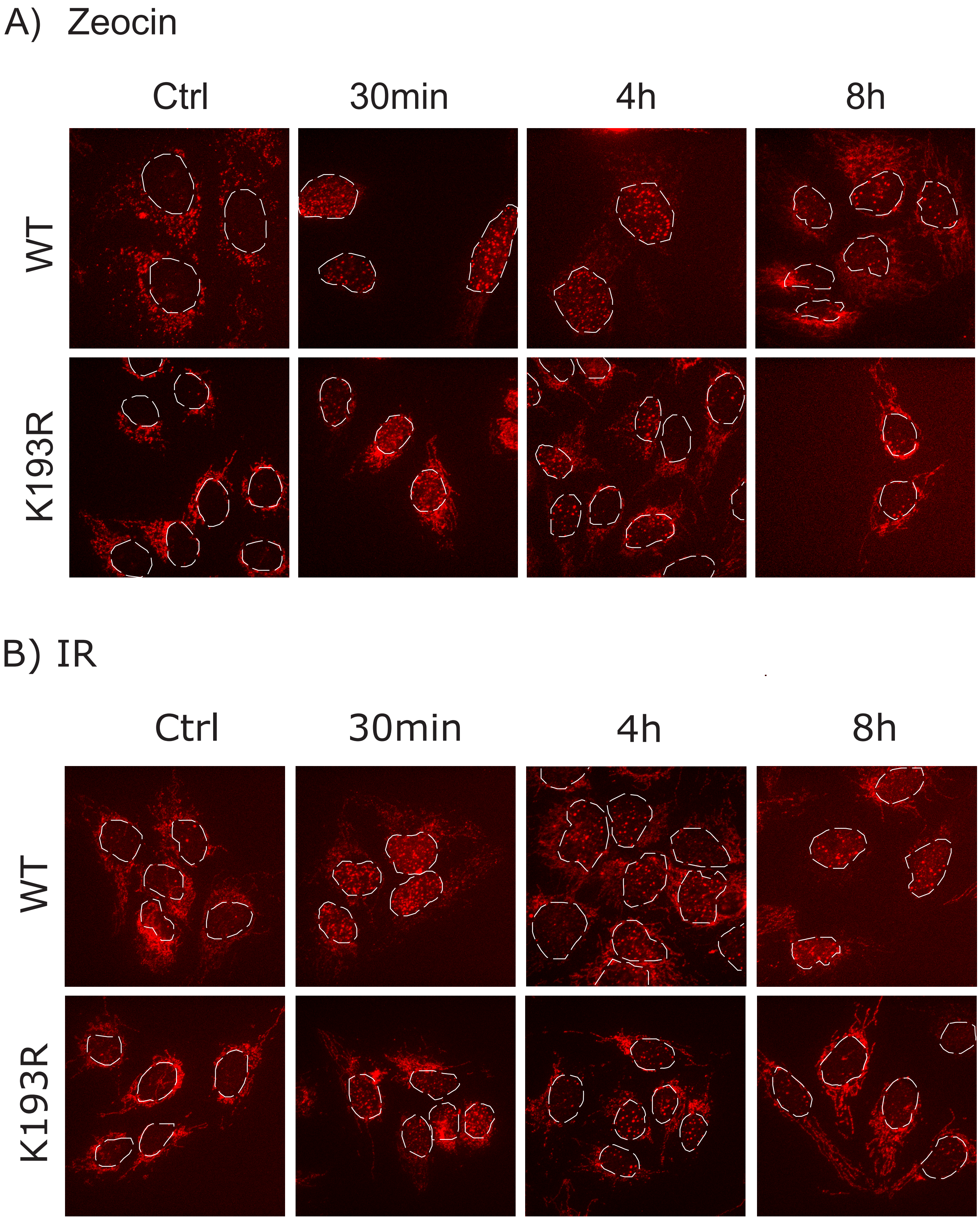
A) Zeocin
Ctrl
30min
4h
8h
WT
K193R
B) IR
Ctrl
30min
4h
8h
WT
K193R

Figure S2.

Table S1: Cryo-EM data collection parameters and statistics.

	Ku70/80 + K193R PAXX peptide<br><br>PDB: 9GYF<br>EMD: 51697
**Data collection and processing**	
Detector	Gatan K3
Magnification	105k
Energy filter slit width (eV)	20
Voltage (kV)	300
Flux on detector (e/pix/sec)	17.1
Electron exposure on sample (e–/Å^2)	1.108
Target defocus range (μm)	0.8-2.0
Calibrated pixel size (Å)	0.824
Symmetry imposed	C1
Extraction box size (pixels)	300
Initial particle images (no.)	1256763
Final particle images (no.)	104184
**Refinement**	
Map resolution at FSC=0.143 (Å)*	2.8
Model composition	
Non-hydrogen atoms	8717
Protein residues	1056
Nucleotides	30
B factor (Å^2)	
Protein	123.78
DNA	175.07
R.m.s deviations	
Bond lengths (Å)	0.003
Bond angles (°)	0.549
Validation	
Molprobity score	1.73
Clashscore	7.18
Poor rotamers (%)	1.18
Ramachandran plot	
Favored (%)	95.90
Allowed (%)	4.10
Disallowed (%)	0.00

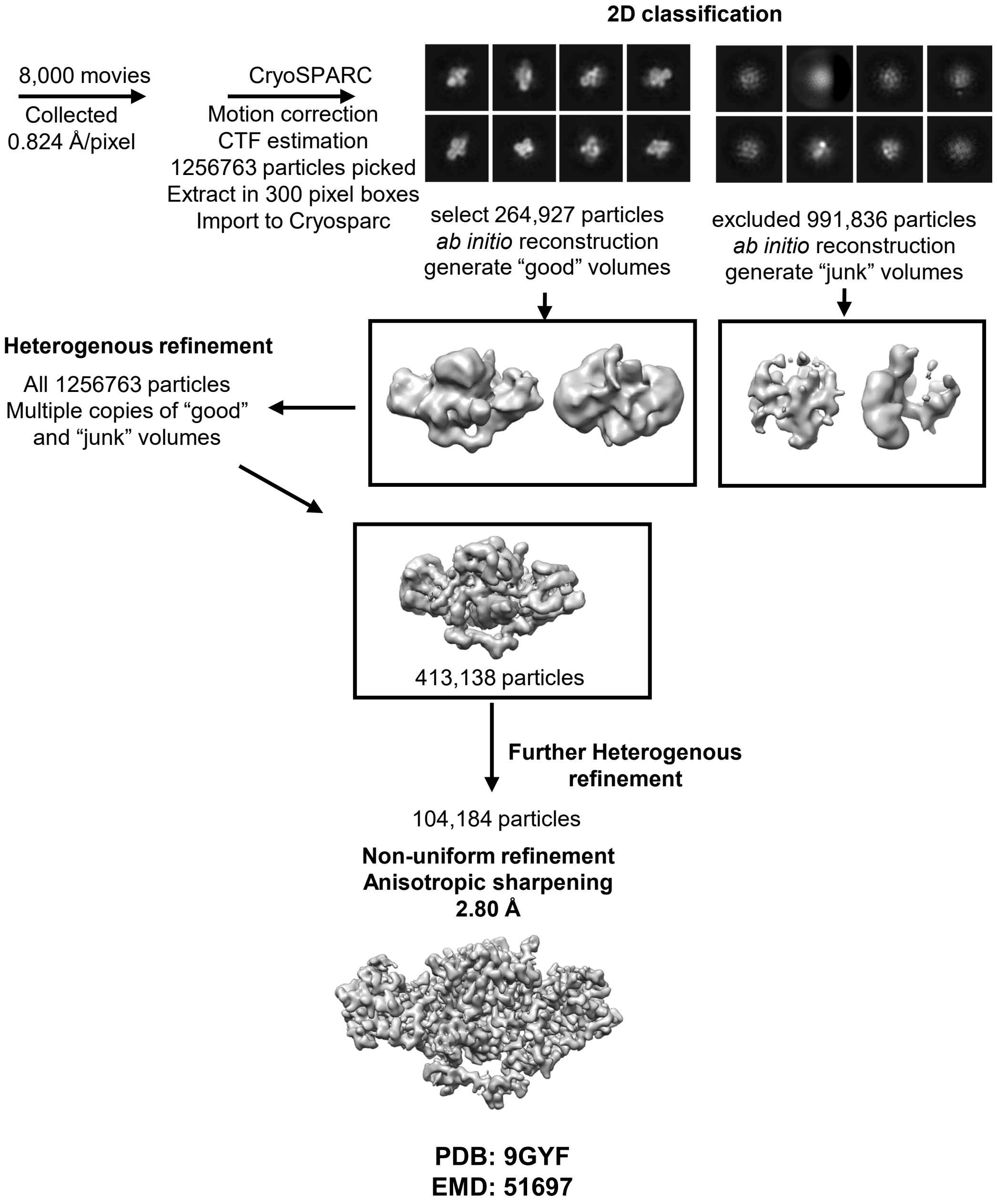
2D classification
8,000 movies
Collected
0.824 Å/pixel
CryoSPARC
Motion correction
CTF estimation
1256763 particles picked
Extract in 300 pixel boxes
Import to Cryosparc
select 264,927 particles
ab initio reconstruction
generate "good" volumes
excluded 991,836 particles
ab initio reconstruction
generate "junk" volumes
Heterogenous refinement
All 1256763 particles
Multiple copies of "good"
and "junk" volumes
413,138 particles
Further Heterogenous
refinement
104,184 particles
Non-uniform refinement
Anisotropic sharpening
2.80 Å
PDB: 9GYF
EMD: 51697

Figure S3.

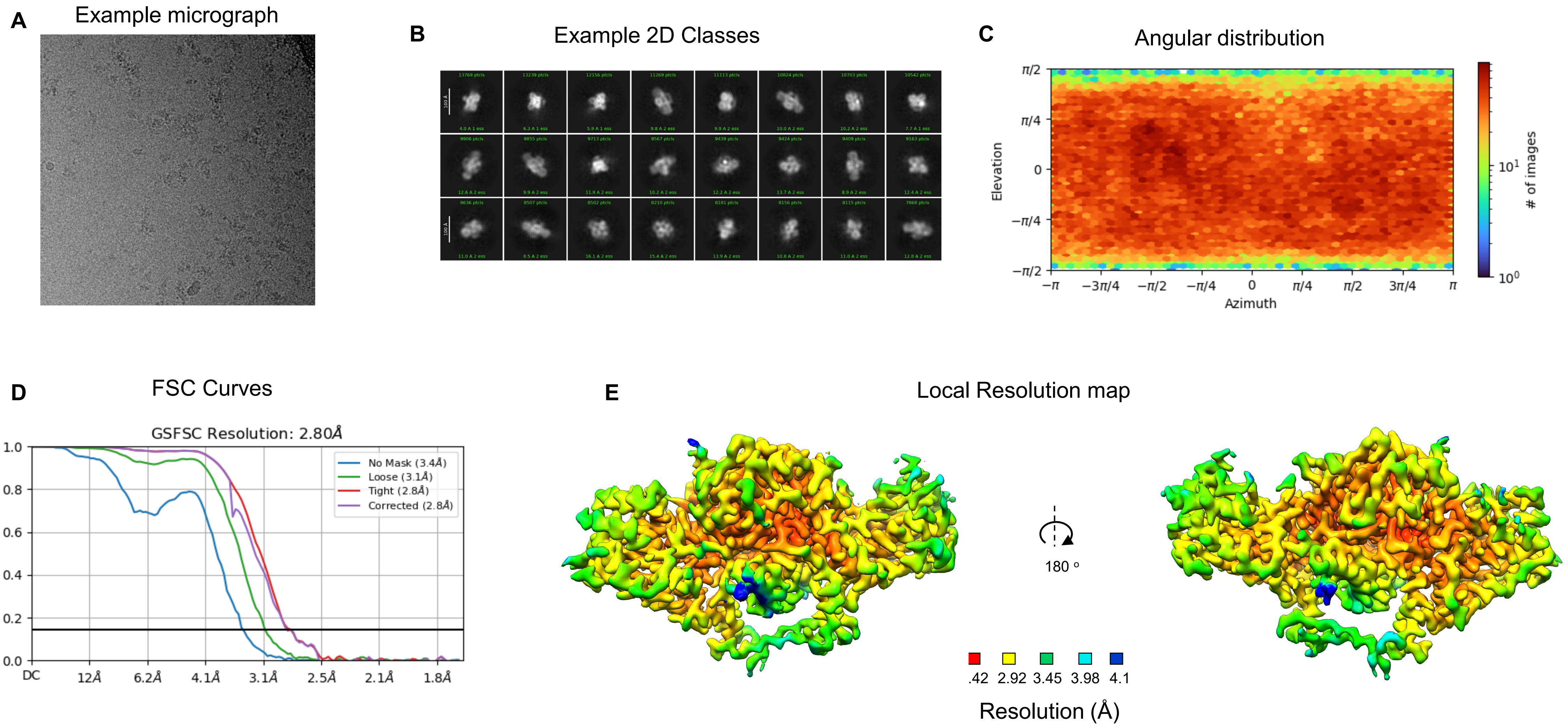
A
Example micrograph
B
Example 2D Classes
C
Angular distribution
Elevation
Azimuth
# of images
D
FSC Curves
GSFSC Resolution: 2.80Å
No Mask (3.4Å)
Loose (3.1Å)
Tight (2.8Å)
Corrected (2.8Å)
DC
12Å
6.2Å
4.1Å
3.1Å
2.5Å
2.1Å
1.8Å
E
Local Resolution map
180 °
.42
2.92
3.45
3.98
4.1
Resolution (Å)

Figure S4.

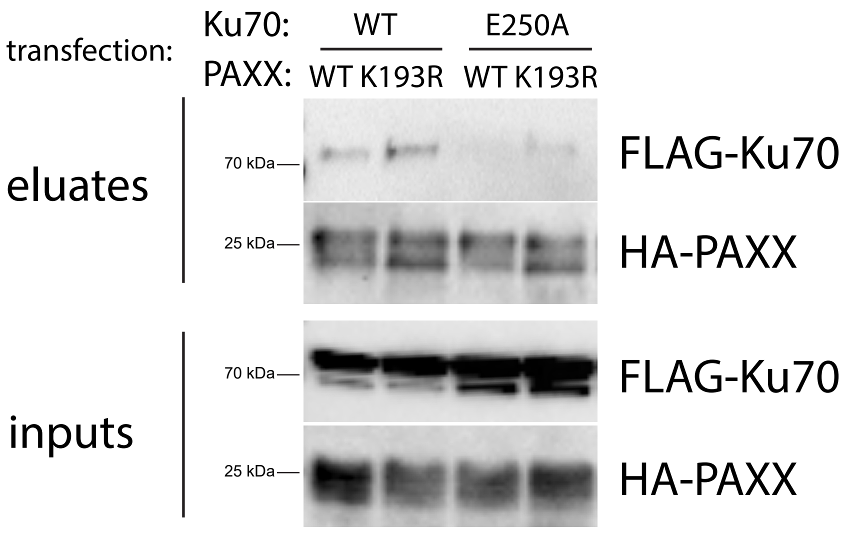
transfection:
Ku70:
WT
E250A
PAXX:
WT K193R WT K193R
eluates
70 kDa
25 kDa
FLAG-Ku70
HA-PAXX
inputs
70 kDa
25 kDa
FLAG-Ku70
HA-PAXX

Figure S5.
